# Utilizing Artificial Intelligence in Cine Magnetic Resonance Imaging Analysis: A Promising Approach for Assessment of Uterine Factors and Prediction of Pregnancy Outcomes in Patients With Recurrent Implantation Failure

**DOI:** 10.1002/rmb2.70028

**Published:** 2026-03-02

**Authors:** Daiki Hiratsuka, Katsuhiko Noda, Kaname Yoshida, Mayu Kinoshita, Yumiko Doi, Okikaze Kato, Kotaro Oshima, Shizu Aikawa, Chihiro Ishizawa, Yamato Fukui, Takehiro Hiraoka, Mitsunori Matsuo, Tomoko Makabe, Gentaro Izumi, Kenbun Sone, Miyuki Harada, Yasushi Hirota

**Affiliations:** ^1^ Department of Obstetrics and Gynecology, Graduate School of Medicine The University of Tokyo Tokyo Japan; ^2^ SIOS Technology, Inc. Tokyo Japan

**Keywords:** artificial intelligence, cine magnetic resonance imaging, infertility, machine learning, peristalsis

## Abstract

**Purpose:**

Recurrent implantation failure (RIF) is a form of refractory infertility that persists despite assisted reproductive technology. Cine magnetic resonance imaging (cine MRI) enables the visualization of uterine peristalsis; however, its use in RIF assessment is limited due to the lack of a standardized application method. This study aimed to develop pregnancy prediction models for patients with RIF and to evaluate the utility of cine MRI image analysis using artificial intelligence (AI).

**Methods:**

We retrospectively analyzed the anonymized clinical data and cine MRI images of 188 patients with RIF and known pregnancy outcomes. Two types of models, based on clinical data only or both clinical data and cine MRI images, were built using the Random Forest model. The best model was identified using the area under the receiver operating characteristic curve (AUC), accuracy, sensitivity, and specificity.

**Results:**

Higher performance was achieved using the Random Forest model integrating clinical data and cine MRI images (AUC/accuracy/sensitivity/specificity: 0.835, 0.754, 0.879, and 0.583, respectively), outperforming models using clinical data only (0.617, 0.596, 0.697, and 0.458, respectively).

**Conclusions:**

AI analysis of clinical data combined with cine MRI data improved pregnancy prediction, suggesting that cine MRI can be used to evaluate uterine factor‐related RIF.

## 1 Introduction

Infertility is a major global health issue affecting approximately 17.5% of couples of reproductive age who wish to conceive [1]. The standard management of infertility generally follows a sequential approach, starting with timed intercourse, progressing to intrauterine insemination (IUI), and eventually to assisted reproductive technology (ART) [2]. Nevertheless, some patients fail to conceive, even after undergoing ART. The main reasons for ART failure can be broadly divided into embryonic factors and uterine factors [3, 4]. While preimplantation genetic testing (PGT) has advanced to detect embryonic abnormalities [5], approaches for identifying and managing uterine factors remain underdeveloped, except for the surgical treatment of clear pathological lesions such as endometrial polyps or uterine fibroids [3, 6, 7, 8]. Clinically, recurrent implantation failure (RIF) is defined as the inability to achieve pregnancy despite the transfer of at least four morphologically good‐quality embryos over three or more cycles in women younger than 40 years old [3]. RIF is regarded as a severe and treatment‐resistant form of infertility, affecting 5%–20% of patients undergoing ART [3, 9]. Establishing effective diagnostic and treatment strategies for RIF is considerably required.

Uterine peristalsis (UP) refers to wave‐like movements that occur in the junctional zone of the nonpregnant uterus in accordance with the menstrual cycle [10]. During the periovulatory phase, these movements occur from the cervix toward the fundus, supporting sperm transport. In contrast, during menstruation, they occur from the fundus toward the cervix, supporting menstrual blood expulsion. Thus, the UP has a physiological goal‐directed function [11]. During the implantation window, the UP decreases because excessive UP can hinder implantation, resulting in reduced pregnancy rates. Consequently, an abnormal UP is recognized as a uterine factor involved in implantation failure [12, 13]. UP was assessed using ultrasound, hysterosalpingography, intrauterine pressure catheters, and cine magnetic resonance imaging (cine MRI) [11]. Specifically, cine MRI enables continuous imaging of the uterus, allowing objective evaluation of peristaltic activity independent of the skill of the examiner. However, despite its ability to visualize the UP with high accuracy, the evaluation of the UP varies depending on the examiner, and no standardized index or model for UP assessment is currently available. Therefore, cine MRI–based evaluation of the UP has not yet been fully incorporated into the clinical practice of reproductive medicine [11, 14].

Artificial intelligence (AI) is a technology that enables computers to perform intellectual tasks on behalf of humans. Machine learning, based on the scientific study of algorithms and statistical models that enable efficient task execution, is a state‐of‐the‐art approach for the development of AI models. By employing an appropriate AI model, computers can learn patterns from the supplied datasets and draw inferences without requiring explicit instructions [15]. Deep neural networks (DNNs), which implement deep learning through multilayered neural architectures, have attracted particular interest in the medical field because of their suitability for image analysis. DNNs are used for the classification, image quality enhancement, and segmentation of medical images [16]. In reproductive medicine, AI models implementing DNN technology have been clinically applied for image‐based evaluation of sperm, embryos, and the ovary [17, 18, 19, 20]. However, no such application has been established for uterine factors, including UP.

In this study, we aimed to develop an AI‐based algorithm to predict pregnancy outcomes using clinical information related to uterine factors combined with cine MRI images and evaluated the utility of AI‐assisted cine MRI image analysis.

## 2 Materials and Method

### 2.1 Data Collection

This study was conducted in accordance with the Declaration of Helsinki and the Ethical Guidelines for Medical and Biological Research Involving Human Subjects established by the Japanese Government. This study was reviewed and approved by the Research Ethics Committee of the Faculty of Medicine at the University of Tokyo (Institutional Review Board No. 10991). Informed consent was obtained from all patients for being included in the study.

Anonymous clinical data were obtained from 188 patients with infertility at the University of Tokyo Hospital who failed to achieve clinical pregnancy after transferring good‐quality embryos (Gardner blastocyst grade ≥ 4BB) in at least two fresh or frozen cycles and underwent cine MRI between June 2016 and July 2023. Clinical information on 15 indices—age; gravidity; parity; number of embryo transfers; complications (adenomyosis, leiomyoma, uterine anomaly, endometrial polyps, oviductal anomaly, or ovarian endometrioma); findings of hysteroscopy; endometrial cluster of differentiation 38 (CD138) test results; vaginal Mycoplasma/Ureaplasma test results and endometrial microbiome test results; number of post‐examination embryo transfers; and clinical pregnancy assessed 1 year after treatment—was extracted from the medical records. Patients with adenomyosis or leiomyomas, those with a uterus larger than the small pelvis, or those with the International Federation of Gynecology and Obstetrics classification II leiomyomas grades 0–3 were excluded from the study. Cine MRI was performed using a 1.5‐T magnet unit (MRI machine from Siemens Medical Systems) with a 6‐ch array coil. Under quiet respiration, 30 serial images were obtained by a single‐shot fast spin‐echo sequence (echo time and repetition time [repetition time/echo time TR/TE] = 6000/39 ms, field of vision = 200 mm, slice thickness = 10 mm, matrix = 256 × 256), every 6 s/3 min in the midsagittal plane of the uterus. All images in one study were summed into one image and displayed sequentially on the cine‐mode display at 250‐ms intervals. Subsequently, conventional axial and sagittal T2‐weighted images (TR/TE = 4000/90–95 ms) and axial T1‐weighted in‐ and out‐of‐phase images (TR/TE = 220/2.21, 4.78 ms) were obtained.

### 2.2 Establishment of the Model Based on Clinical Data Only

In the clinical‐only model, patient‐level clinical features were used as predictors, and the pregnancy outcome label (truth) was used as the target. The 15 baseline variables described in the *Data collection* section included age, gravidity, parity, number of embryo transfers, and number of post‐examination embryo transfers as continuous variables. All others were encoded as binary indicators (1 = lesion present or test positive, 0 = normal). Two composite variables were also created: pathology count (sum of binary indicators for adenomyosis, leiomyoma, uterine anomaly, endometrial polyps, oviductal anomaly, and ovarian endometrioma) and inflammation score (sum of hysteroscopy, endometrial CD138, vaginal *Mycoplasma/Ureaplasma*, and endometrial microbiome test results). Missing values were median‐imputed within the training folds, and infinite or invalid entries were similarly replaced.

To prevent patient‐level leakage and maintain class balance, data were split using *GroupShuffleSplit* (70% training, 30% validation; random seed = 42), ensuring that all data from a given patient were allocated to a single partition. To verify that this split resulted in comparable groups, statistical comparisons of the variables were conducted using Fisher's exact test for categorical variables and Student's *t*‐test for continuous variables, implemented in R software (version 4.3.3).

Within the training set, *GroupKFold* (five folds) with *GridSearchCV* was applied to optimize *RandomForestClassifier* hyperparameters for accuracy. Model performance on the validation set was assessed using the area under the curve‐receiver operating characteristic (ROC‐AUC), accuracy, sensitivity, specificity, and balanced accuracy (the mean of sensitivity and specificity). Confusion matrices derived from binary pregnancy outcomes (success vs. failure) were used to calculate sensitivity and specificity.

### 2.3 Establishment of DNN Models for Cine MRI

#### 2.3.1 Variation of Image Types for Training and Evaluation

**FIGURE 1:**
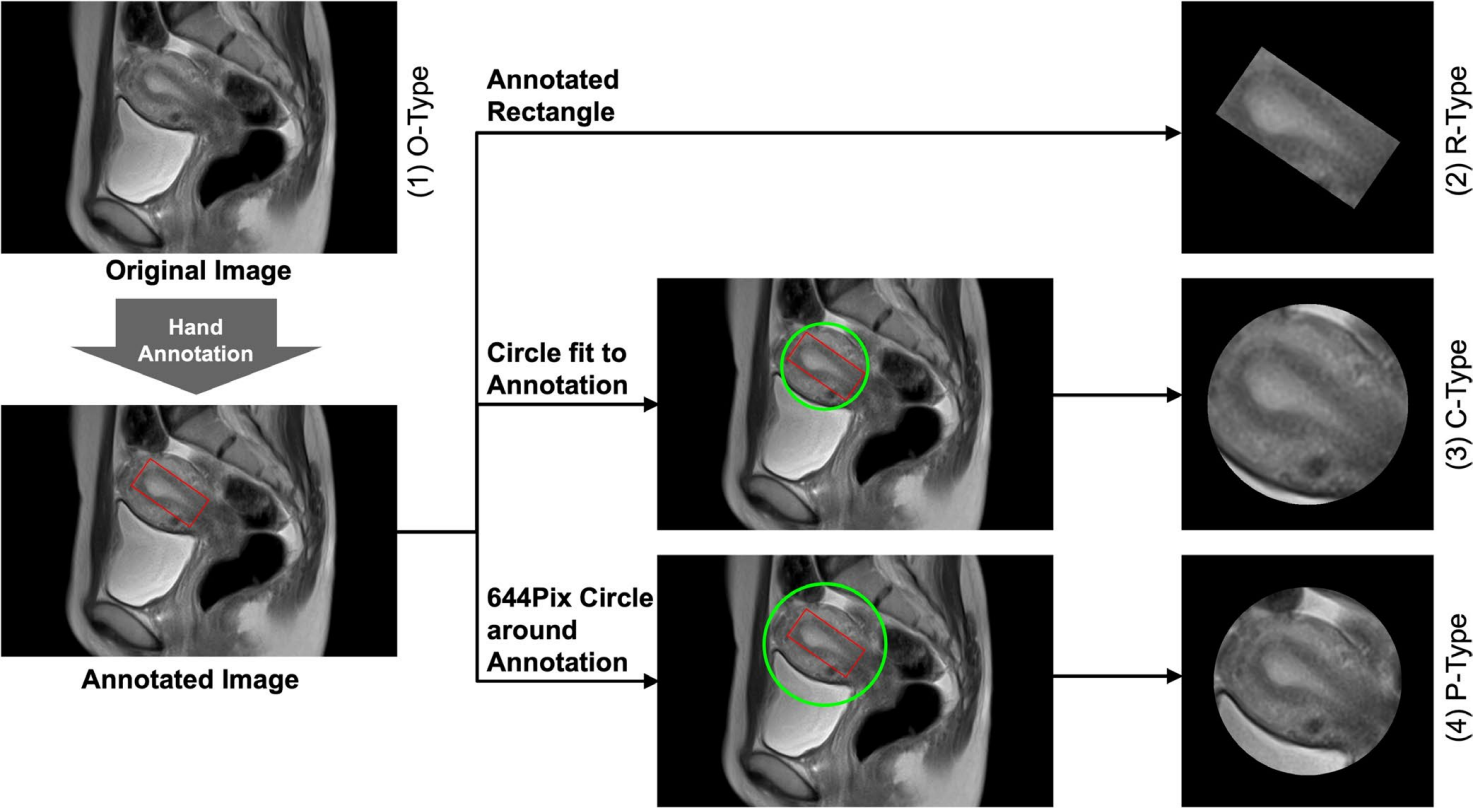
Concept of the four types of magnetic resonance imaging (MRI) images used for deep neural networks. The red rectangle represents the annotation by hand. Green circles were added depending on the red rectangles. Four types (O‐type, R‐type, C‐type, and P‐type) were prepared.

We hypothesized that the DNN accuracy would improve when trained on MRI regions limited to the uterus. To extract these regions, a gynecologist manually annotated the uterus on each MRI slice by enclosing it within a red rectangle. Based on these annotations, four image types were prepared for training and evaluation to compare performance differences (Figure 1).

1. O‐type: Original entire MRI images
2. R‐type: Images cropped from annotated rectangles
3. C‐type: Images cropped from a circumscribed circle of a rectangle
4. P‐type: Images cropped from a fixed 644‐pixel‐diameter circle centered on the rectangle.

#### 2.3.2 Method for Training DNN to Learn UP

3D CNN models are generally used to train DNNs to learn the motions in videos [21]. However, because 3D CNNs require high computational costs and large datasets, we adopted a 2D CNN instead. To use a 2D CNN, the features of the UP in cine MRI must be converted into a two‐dimensional form. We developed an original method combining four consecutive grayscale frames (f1–f4) into one RGB image (R, G, B) using the following formula:

$$\left[R,G,B\right]=\left[f1/2-f2/2+128,f2/2-f3/2+128,f3/2-f4/2+128\right]$$

**FIGURE 2:**
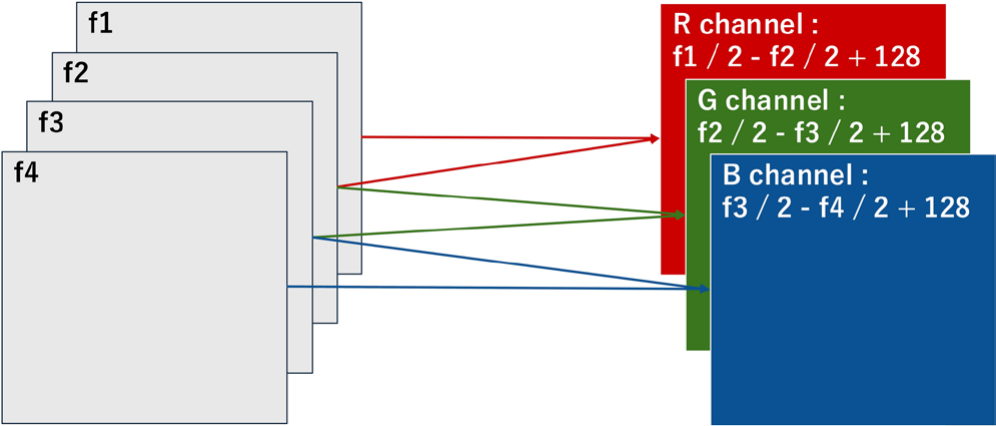
Concept of our method to extract features of uterine peristalsis (UP). Four consecutive grayscale images (f1, f2, f3, and f4) were combined into one color image (R, G, and B) that represented the UP for 18 s.

Each RGB image represented the uterine motion over 18 s (Figure 2). We employed MobileNet‐V2, which is a compact DNN with 88 layers, an input size of 224 × 224 pixels, and 3 538 984 trainable parameters.

#### 2.3.3 Training of DNN Models for Cine MRI

Six‐fold cross‐validation was used to divide all cases into six groups: five for training and one for evaluation (5:1). Each patient was never included in the training set when their prediction was made, thereby preventing any patient‐level data leakage. Accordingly, six DNN models were built for each of four image types (O, R, C, and P‐type). The training images were augmented 40‐fold, and in each epoch, one‐twentieth of the augmented data was sampled randomly. The training was repeated for 20 epochs per cross‐validation set. Because the model performance varied owing to the stochastic effects of augmentation, 12 independent repetitions of 6‐fold cross‐validation were performed to assess the performance variability. Thus, 288 models were generated (6 × 12 × 4 = 288).

#### 2.3.4 Evaluation of DNN Models for Cine MRI

The model evaluation was conducted using two approaches: image‐unit‐based and case‐unit‐based, for all four image types.

For image‐unit evaluation, predictions were made by (1) single models and (2) ensemble averaging of 11 models derived from 12 independent repetitions of 6‐fold cross‐validation. For case‐unit evaluation, the prediction scores were summed across 20 consecutive cine MRI frames (2 min; 17 combined images) for 10 pairs per case, and the pair with the highest total score was used as the representative value to predict implantation success or failure. This evaluation was also performed in both single‐model and ensemble modes for the four image types.

### 2.4 Establishment of the Model Based on Both Clinical Data and Cine MRI Images

The same 188 patients used in the clinical‐only analysis were analyzed using the cine MRI ensemble scores appended to the clinical feature set. In the Section 3, we report that the P‐type ensemble model achieved the highest predictive performance among all image types; therefore, P‐type ensemble scores were used for clinical integration. For each patient, 12 cine MRI ensemble records were summarized into five statistical descriptors: “Score_mean”, “Score_std” (standard deviation), “Score_range”, “Score_IQR” (interquartile range), and “Score_median”, and merged with the clinical dataset. The combined feature matrix contained 22 predictors: 17 clinical variables (15 baseline clinical indices plus Pathology_count and Inflammation_score) and five cine MRI summary statistics. Missing or infinite cine MRI values were median imputed, and undefined Score_std entries were set to zero. Clinical preprocessing followed the same workflow as that of the clinical‐only model.

**FIGURE 3:**
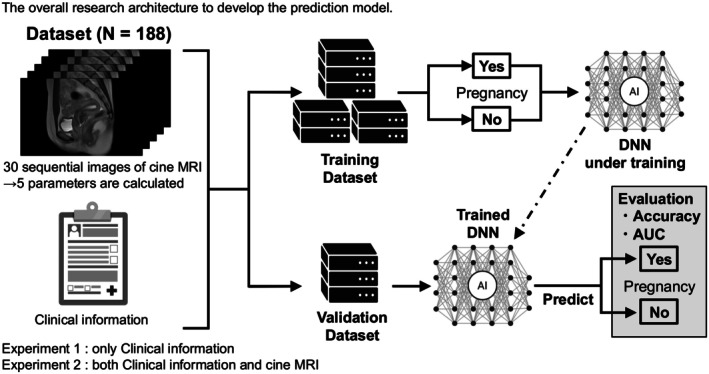
Overall architecture of the development of the model in this study.

To prevent patient‐level leakage, data were split using GroupShuffleSplit (70% training, 30% validation; random seed = 42). Within the training set, GroupKFold (five folds) with GridSearchCV was applied to optimize the random forest classifier hyperparameters for accuracy. Model performance on the validation set was evaluated using ROC‐AUC, accuracy, sensitivity, specificity, and balanced accuracy (the mean of sensitivity and specificity). Confusion matrices derived from binary pregnancy outcomes (success vs. failure) were used to calculate sensitivity and specificity. The overall architecture of the model, based on both clinical data and cine MRI images, is shown in Figure 3.

## 3 Results

### 3.1 Patient Characteristics and Feature Values for the Machine Learning Dataset

In total, 188 patients with RIF were enrolled in this study. Table 1 summarizes the patient characteristics and feature values used for the machine learning. Of 188 patients, 55.3% (*n* = 104/188) got pregnant after the examination. No statistically significant differences in clinical information were observed between the train and validation datasets, confirming that the patient characteristics were balanced and suitable for model training and evaluation (Table 2).

**TABLE 1:** Patients' clinical information.

Characteristics	Mean ± Standard deviation
Age	36.8 ± 4.1
Gravidity	0.6 ± 1.0
Parity	0.2 ± 0.4
The number of embryo transfers (before the tests)	3.7 ± 2.3
The number of embryo transfers (after the tests)	1.6 ± 0.9
Leiomyoma	38.3% (*n* = 72/188)
Adenomyosis	17.6% (*n* = 33/188)
Ovarian endometrioma	20.2% (*n* = 38/188)
Endometrial polyp	1.6% (*n* = 3/188)
Uterine anomaly	5.3% (*n* = 10/188)
Oviductal anomaly	2.1% (*n* = 4/188)
Abnormal hysteroscopic findings	50.5% (*n* = 94/188)
Endometrial cluster of differentiation 138 (CD138) positivity	42.9% (*n* = 78/182)
Non‐lactobacillus dominant microbiota	59.5% (*n* = 25/42)
Vaginal micoplasma/ureaplasma positivity	20.0% (*n* = 31/155)
Pregnancy (Gestational Sac)	55.3% (*n* = 104/188)

**TABLE 2:** Comparison of clinical characteristics between the training and validation datasets.

Characteristics	Training dataset (*n* = 131)	Validation dataset (*n* = 57)	*p*
Age	36.4 ± 4.3	37.7 ± 3.6	0.058
Gravida	0.5 ± 0.8	0.8 ± 1.3	0.125
Para	0.1 ± 0.3	0.2 ± 0.4	0.161
The number of embryo transfer (before the tests)	3.6 ± 2.3	3.8 ± 2.3	0.665
The number of embryo transfer (after the tests)	1.5 ± 0.8	1.7 ± 1.1	0.199
Leiomyoma	37.4% (*n* = 49/131)	40.4% (*n* = 23/57)	0.745
Adenomyosis	16.0% (*n* = 21/131)	21.1% (*n* = 12/57)	0.411
Ovarian endometrioma	18.3% (*n* = 24/131)	24.6% (*n* = 14/57)	0.33
Endometrial polyp	0.8% (*n* = 1/131)	3.5% (*n* = 2/57)	0.219
Uterine anomaly	4.6% (*n* = 6/131)	7.0% (*n* = 4/57)	0.494
Oviductal anomaly	2.3% (*n* = 3/131)	1.8% (*n* = 1/57)	1.000
Abnormal hysteroscopic findings	49.6% (*n* = 64/129)	52.6% (*n* = 30/57)	0.752
Endometrial CD138 positivity	40.0% (*n* = 50/125)	49.1% (*n* = 28/57)	0.262
Non‐lactobacillus dominant microbiota	59.4% (*n* = 19/32)	60.0% (*n* = 6/10)	1.000
Vaginal micoplasma/ureaplasma positivity	17.9% (*n* = 19/106)	24.5% (*n* = 12/49)	0.390
Pathology count	0.8 ± 0.8	1.0 ± 1.0	0.176
Inflammation score	1.2 ± 0.9	1.3 ± 0.9	0.217
Pregnancy (Gestational Sac)	54.2% (*n* = 71/131)	57.9% (*n* = 33/57)	0.75
Cine MRI–derived statistical descriptors			
Score_mean	0.60 ± 0.24	0.55 ± 0.26	0.532
Score_std	0.02 ± 0.01	0.02 ± 0.01	0.520
Score_range	0.07 ± 0.02	0.06 ± 0.02	0.874
Score_IQR	0.03 ± 0.02	0.03 ± 0.01	0.738
Score_median	0.57 ± 0.23	0.60 ± 0.22	0.376

### 3.2 Predictive Performance of the Clinical‐Only RandomForest Model

**FIGURE 4:**
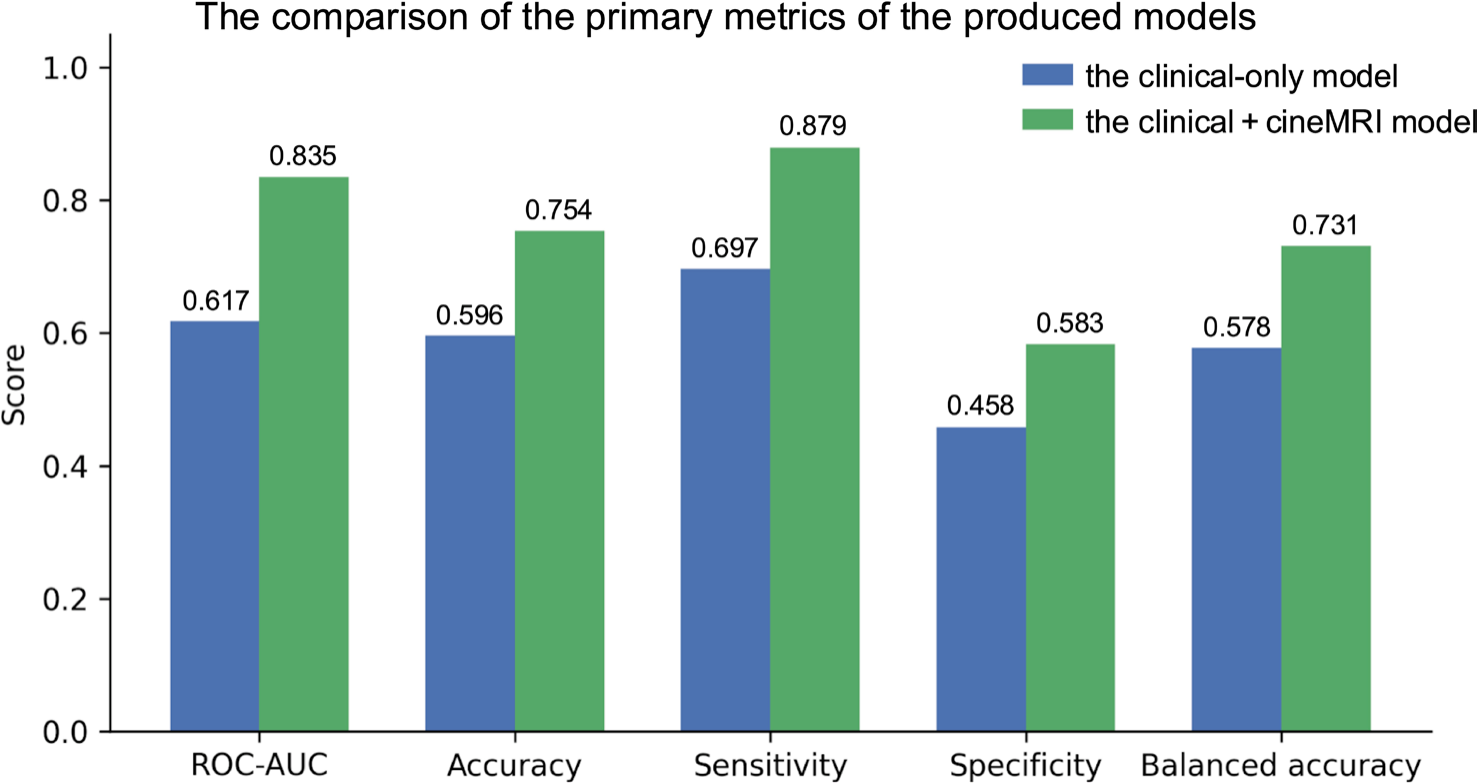
Comparison of the primary metrics for the clinical‐only model and the clinical + cineMRI statistical configurations model.

The clinical‐only RandomForest achieved an ROC‐AUC of 0.617, accuracy of 0.596, sensitivity of 0.697, specificity of 0.458, and balanced accuracy of 0.578 in the validation cohort (Figure 4).

### 3.3 Establishment of DNN Models for Cine MRI

Table 3 presents the results of the image‐unit‐based evaluations. Each number represents the mean ± standard deviation of the predictions made by the 12 single DNN models or 12 pairs of ensembles with 11 models. This result demonstrates that the ensemble prediction on P‐type (images cropped 644‐pixel diameter circle centered on the annotated rectangle) images achieved the best accuracy of 64.55% ± 0.17% (area under the receiver operating characteristic curve [AUC] = 68.27% ± 0.12%, sensitivity = 73.44% ± 2.66%, specificity = 55.65% ± 2.65%). Notably, the ensemble models showed smaller standard deviations compared to the single models, indicating improved prediction stability across runs. Table 4 presents the results of the case unit‐based evaluation. Each number represents the mean ± standard deviation of the predictions made by the 12 single DNN models or 12 pairs of ensembles with 11 models. This result demonstrates that the ensemble prediction on P‐type (images cropped 644‐pixel diameter circle centered on the annotated rectangle) images achieved the best accuracy of 68.89% ± 0.50% (AUC = 69.22% ± 0.25%, sensitivity = 76.28% ± 2.97%, specificity = 61.51% ± 3.26%), again showing reduced variability with ensemble predictions, supporting the robustness of the ensemble approach.

**TABLE 3:** Result of image‐unit‐based evaluation of cine MRI (mean ± standard deviation).

Image data	Ensemble	AUC	Sensitivity	Specificity	Accuracy
O‐type	Ensemble	62.24% ± 0.23%	46.02% ± 2.15%	72.31% ± 2.02%	59.16% ± 0.28%
	Single	60.43% ± 1.95%	45.08% ± 12.39%	70.66% ± 11.57%	57.87% ± 1.40%
R‐type	Ensemble	48.86% ± 0.22%	8.78% ± 19.69%	93.65% ± 19.79%	51.22% ± 0.11%
	Single	49.08% ± 1.88%	57.97% ± 43.65%	45.41% ± 43.21%	51.69% ± 0.77%
C‐type	Ensemble	56.50% ± 0.26%	36.74% ± 19.34%	73.53% ± 19.21%	55.14% ± 0.18%
	Single	55.73% ± 2.71%	34.55% ± 19.76%	75.98% ± 19.61%	55.27% ± 1.96%
P‐type	Ensemble	68.27% ± 0.12%	73.44% ± 2.66%	55.65% ± 2.65%	64.55% ± 0.17%
	Single	63.82% ± 0.98%	70.07% ± 8.71%	51.07% ± 8.61%	60.57% ± 0.49%

**TABLE 4:** Result of case‐unit‐based evaluation of cine MRI (mean ± standard deviation).

Image Data	Ensemble	AUC	Sensitivity	Specificity	Accuracy
O‐type	Ensemble	59.54% ± 0.34%	77.80% ± 10.44%	41.17% ± 10.73%	59.49% ± 0.46%
	Single	58.09% ± 2.39%	59.86% ± 23.50%	56.85% ± 23.33%	58.35% ± 1.34%
R‐type	Ensemble	50.74% ± 0.21%	66.19% ± 12.58%	40.97% ± 12.70%	53.58% ± 0.52%
	Single	50.56% ± 1.39%	66.11% ± 24.39%	42.46% ± 24.35%	54.28% ± 0.93%
C‐type	Ensemble	54.81% ± 0.27%	43.59% ± 4.09%	71.83% ± 4.13%	57.71% ± 0.46%
	Single	53.93% ± 2.42%	45.99% ± 23.58%	65.67% ± 23.13%	55.83% ± 1.37%
P‐type	Ensemble	69.22% ± 0.25%	76.28% ± 2.97%	61.51% ± 3.26%	68.89% ± 0.50%
	Single	64.44% ± 1.40%	77.40% ± 8.87%	49.01% ± 8.86%	63.21% ± 1.31%

### 3.4 Predictive Performance of the Random Forest Model Integrating Clinical Data and Cine MRI Images

The five incorporated cine MRI–derived statistical descriptors showed no significant differences between the training and validation datasets, ensuring a consistent distribution of image‐based features across the split (Table 2). Incorporating cine MRI statistics increased RandomForest performance to an ROC‐AUC, accuracy, sensitivity, specificity, and balanced accuracy of 0.835, 0.754, 0.879, 0.583, and 0.731, respectively (Figure 4). Sensitivity increased by 0.182 points and specificity increased by 0.125 points, indicating improved discrimination for both positive and negative outcomes.

## 4 Discussion

In this study, we developed pregnancy prediction models for patients with RIF and evaluated the utility of AI‐based cine MRI image analysis. Our results demonstrate that DNN models integrating clinical and cine MRI data outperform those based on clinical data alone in predicting pregnancy outcomes. The clinical use of cine MRI and UP evaluation in RIF remains limited; however, our results highlight that AI‐assisted UP analysis has the potential to enhance diagnostic precision and broaden the role of cine MRI in reproductive assessment.

UP decreases during the luteal phase, and abnormal UP has been linked to implantation failure [10, 11, 12, 13]. However, no consensus is available on the optimal modality or parameters to evaluate UP, nor a clear definition of “normal” UP that supports implantation. UP is typically evaluated using parameters such as contraction frequency, amplitude, direction, and coordination [22]; however, most studies have assessed these features in isolation. A comprehensive evaluation using integrated indices has not been widely adopted. A unique aspect of our study is that, by applying a DNN, we were able to incorporate and assess all these factors simultaneously.

In practice, the UP is assessed using imaging modalities such as ultrasound or cine MRI. However, these images also contain other information, including uterine size, the presence of fibroids or adenomyosis, and uterine orientation, which may influence UP. Therefore, predictive models must leverage the full range of information embedded in images to improve the accuracy of predicting pregnancy outcomes. Machine learning‐based image analysis is well‐suited to this task because it can integrate such multifactorial data. We employed an original technique to convert four sequential grayscale MRI frames into a single RGB image reflecting the uterine motion over 18 s. While 3D CNNs are generally suitable for spatiotemporal learning, they require significantly larger datasets and incur higher computational costs. Given the limited frame rate and sample size of clinical cine MRI, we opted for a 2D CNN (MobileNet‐V2) and achieved robust performance through data augmentation and ensemble learning. Among the four evaluated image types (O‐type, R‐type, C‐type, and P‐type), the P‐type ensemble model exhibited the best predictive performance. This may be due to its standardized circular field of view, which preserves critical uterine motion patterns while minimizing irrelevant backgrounds and anatomical variability. The stable and superior performance of the p‐type model supports its suitability for learning uterine dynamics in a clinically consistent manner. Additionally, we maximized UP evaluation by combining clinical data with cine MRI (approximately 30 frames per case) in our predictive model.

Regarding therapeutic interventions for an abnormal UP, agents such as oxytocin receptor antagonists and anticholinergics have been explored [23, 24, 25, 26], and myomectomy for fibroids associated with an abnormal UP has been reported to improve peristalsis and increase pregnancy rates [27, 28]. These findings highlight the clinical significance of treating patients with abnormal UP levels. Nonetheless, no established therapy exists for UP abnormalities, and the lack of a standardized UP evaluation has hampered the development of targeted interventions. This study is the first to demonstrate that AI‐based cine MRI analysis can improve the precision of UP evaluations. With the development of more stable and highly accurate models, such approaches may establish UP assessment as a reliable diagnostic tool and advance therapies for implantation failure.

This study had some limitations. First, the sample size was relatively small, as patients with RIF undergoing cine MRI were limited. We performed all cine MRI scans under standardized luteal‐phase conditions and excluded cases with confounding factors (including large pelvic fibroids or L0–3 fibroids) to create a more homogeneous cohort for UP analysis, thus ensuring study quality. As more cine MRI cases are accumulated and integrated into the learning model, the predictive accuracy is expected to improve. Secondly, we included only a limited subset of uterine pathologies (small fibroids, endometrial polyps, and focal adenomyosis) because our primary objective was to evaluate the utility of cine MRI‐based UP assessment. Additionally, abnormal endometrial microbiota were tested in only a few patients, resulting in many missing values [29]. Having demonstrated the value of AI‐assisted cine MRI analysis for UP, future research should extend to broader uterine pathologies to develop models capable of comprehensive uterine evaluation beyond UP alone. Third, we could not use a 3D CNN model. We constructed a highly accurate prediction model by combining two‐dimensional image frames; however, future studies should validate these findings using 3D CNNs. Fourth, this study did not include an independent external test set. Although we used repeated cross‐validation and a held‐out validation set to avoid data leakage and overfitting, external validation on an independent dataset is essential to assess model generalizability and promote future clinical application.

In conclusion, we developed a pregnancy prediction model for patients with RIF by applying a DNN to cine MRI images in combination with clinical data. Our results demonstrated the utility of UP assessment and highlighted the feasibility of AI‐based modeling in this context. These findings suggest that cine MRI, when combined with AI analysis, offers substantial potential as a novel, noninvasive tool for evaluating and managing uterine factors contributing to RIF.

## Funding

This research was supported by JSPS (Grant Numbers JP23K15827, JP24K23524, JP23K27176, JP23K24481, JP24K22157, JP23K23803, and JP24K21911), AMED (Grant Numbers JP24gn0110085, JP24gn0110069, JP24gk0210039, and JP24lk0310083), JST (Grant Number JPMJFR210H), and the Children and Families Agency (Grant Number JPMH23DB0101).

## Ethics Statement

The study was conducted in accordance with the Declaration of Helsinki and the Ethical Guidelines for Medical and Biological Research Involving Human Subjects established by the Japanese Government. This study was reviewed and approved by the Research Ethics Committee of the Faculty of Medicine at the University of Tokyo (Institutional Review Board No. 10991).

## Consent

Informed consent was obtained from all patients for being included in the study.

## Conflicts of Interest

The authors declare no conflicts of interest.

## Data Availability

The data that support the findings of this study are available from the corresponding author upon reasonable request.
